# Identification of novel genetic mutations for the treatment prognostication of canine lymphoma

**DOI:** 10.1038/s41698-025-00988-5

**Published:** 2025-06-12

**Authors:** Josephine Tsang, Qi Jing Yap, Sheena Kapoor, Jerry Cromarty, Sushmita Sen, Minji Kim, George Courcoubetis, Suhyeon Cho, Deanna Swartzfager, Stanley Park, Sungwon Lim, Ilona Holcomb, Jamin Koo

**Affiliations:** 1 ImpriMed, Inc., Mountain View, CA USA; 2 ImpriMedKorea, Inc., Seoul, Republic of Korea; 3 Department of Chemical Engineering, Hongik University, Seoul, Republic of Korea

**Keywords:** Cancer genetics, Lymphoma, Prognostic markers

## Abstract

Canine lymphoma, a phenotypically and genetically heterogeneous disease, represents a significant proportion of canine cancers. We present a large-scale study of 238 dogs with lymphoma to better understand the genetic landscape of canine lymphoma, as well as the relationship to clinical outcomes. Using a targeted next-generation sequencing panel comprising 308 genes, we screened somatic and germline mutations in matched tumor and normal samples. Our findings revealed key associations between genetic alterations and lymphoma subtypes, with certain somatic variants linked to significant differences in response to common chemotherapy regimens. Recurrent mutations in genes such as KMT2C, KMT2D, NOTCH2, TRAF3, CCND1, ARID1A, CREBBP, and TP53 were observed, with TRAF3 mutations standing out for their significant association with prolonged progression-free survival and overall survival in B-cell lymphomas. In contrast, mutations in PIK3CD and CREBBP were associated with inferior outcomes in T-cell lymphomas, highlighting the immunophenotype-specific impact of genetic alterations on treatment responses. These findings support the integration of comprehensive genomic profiling in planning treatment strategies and optimizing clinical outcomes in canine lymphomas.

## Introduction

Canine lymphoma shows striking similarities to human non-Hodgkin lymphoma^1^ and is a disease marked by its phenotypic, clinical, and genetic heterogeneity. This neoplastic condition constitutes a significant part of canine cancers, representing 6% of all cancers and 90% of hematopoietic malignancies^2,3^. Given the known roles of certain cancer-related genes in human oncology, we hypothesized that somatic mutations found in genes of functionally relevant pathways—such as DNA damage response, immune signaling, or cell cycle regulation—may correlate with clinical outcomes in dogs with lymphoma.

Genetic mutations play a fundamental role in cancer biology, influencing tumorigenicity, disease progression, and clinical responses to chemotherapeutic drugs. A recent study has identified potential prognostic biomarkers across canine cancers, noting that somatic mutations in genes with established roles in human cancer biology, such as CCND1, SMARCB1, FANCG, CDKN2B, MSH6, and CCND3, correlate with shorter progression-free survival (PFS) in pan-cancer studies of canine patients^4^. Previous studies in canine lymphoma subtypes have predominantly focused on B-cell lymphoma (BCL), identifying recurrent somatic mutations in TRAF3 and MAP3K14, as well as associations between mutations in POT1, MYC, and TP53 with shorter PFS and overall survival (OS) in dogs with diffuse large B-cell lymphoma (DLBCL)^5^. However, the clinical relevance and consequences of genetic alterations in T-cell lymphoma (TCL) remain largely unexplored, underscoring the need for deeper investigation.

Human oncology has benefited from the development of gene panels for targeted therapy and prognostication. Gene panels employing targeted next-generation sequencing (tNGS) technology, notably the Oncomine panel and the MSK-IMPACT panel, have received FDA approval for both in pan-cancer and cancer type-specific contexts^6,7^. Genetic screenings in canine oncology are also increasing, with targeted mutation analysis in genes such as C-KIT and BRAF increasingly guiding clinical decisions. However, the routine use of comprehensive cancer hotspot panels in dogs, akin to those in human oncology, remains unfulfilled^8,9^. There is a pressing need for a systematic approach to integrate genomic information into everyday veterinary clinical practice.

Integration of NGS technology into clinical practice faces unique challenges, particularly in veterinary oncology. A crucial aspect of this integration involves establishing robust relationships between genotypes of neoplastic conditions and phenotypic characteristics. There is also variability in treatment regimens across different veterinary practices, contributing to a scarcity of data linking genetic mutations to specific treatment outcomes. This study aims to address these gaps through a large-scale analysis of 238 dogs with lymphoma. Each case includes matched tumor and normal samples, which have been screened using a tNGS panel comprising 308 genes pertinent to cancer biology and pharmacogenomics curated from the COSMIC database^10^ and literature^11–13^. We investigate both somatic and germline single-nucleotide variants (SNVs) and insertion-deletion (INDEL) mutations, comparing mutational profiles and immune subtypes against treatment outcomes.

## Results

### Study population and characteristics

The cohort of 238 dogs that met the inclusion criteria for this retrospective study was selected from a pool of 4256 client-owned dogs treated for lymphoma between 2017 and 2023. A summary of the patient demographics, immunophenotypic distribution, and the observed survival characteristics for first-line treatment can be found in Table 1. The incidence rates of BCL and TCL observed in this study reflect those of the general canine population^14^. BCL cases predominantly involved medium (69/153, 45%) and large-cell lymphoma (72/153, 47%). Within TCL cases, the CD4+ T-helper subset was the most prevalent (60/83, 72%). The most represented breed in the study cohort was Labrador Retriever (30/238, 13%), followed by Boxer (26/238, 11%) and Golden Retriever (25/238, 11%). Among the remaining 158 dogs, 79% were purebred, representing 49 different breeds, while 12% were of mixed or unknown breed (Supplementary Table 1). This study documented 433 responses to various regimens consisting of one to five drugs (Supplementary Fig. 1). Monotherapy regimens were the most frequently observed treatment group, accounting for 41%, followed by CHOP and COP-like regimens (38%), MOPP-based regimens (9%), double-agent regimens (6%), and LOPP-based regimens (4%).

**Table 1:** Baseline characteristics of the lymphoma cohort

Characteristic	*N* = 238
Sex	
Male	11
Male neutered	116
Female	2
Female spayed	104
Unknown	5
Age at diagnosis, years	9 (2–18)
Clinical stage	
I	1
II	14
III	99
IV	57
V	24
Not staged	43
Immunophenotype	
BCLs	153
TCLs	83
Other	2
Survival, days	
Median PFS	102
Median OS	277

### Mutational landscape of canine lymphoma

**Fig. 1:**
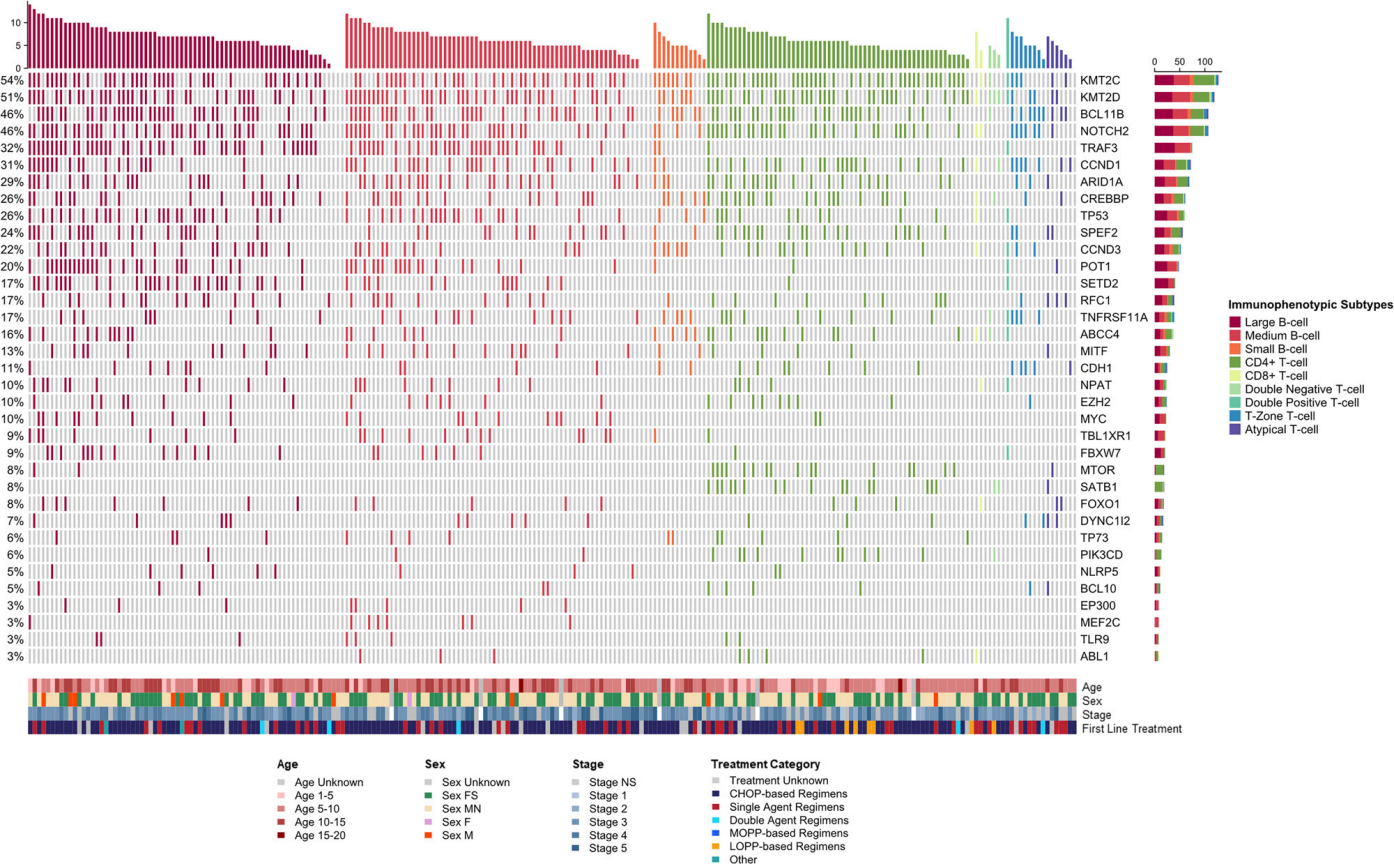
Oncoplot depicting somatic mutations of the top 35 genes in 238 dogs, segmented by the major immunophenotypic subtypes of canine lymphoma. Demographic characteristics of our cohort are shown in a secondary heatmap.

We evaluated the most common somatic mutations observed in relation to patient signalment, treatment status (treatment-naive or relapsed), stage, and immunophenotype (Fig. 1). Mutation profiles did not differ significantly for patient sex (*P* > 0.4), age (*P* > 0.9), or stage of the disease (*P* > 0.9). However, we did note several mutated genes associated significantly with certain immunophenotypes (Supplementary Fig. 2). In BCLs, TRAF3 ranked as the third most frequently mutated gene, showing a higher mutation frequency in CD21+ DLBCL and a lower occurrence in CD4+ TCL (*P* < 0.001). FBXW7 and POT1 were also significantly associated with DLBCLs (*P* < 0.05), aligning with previous studies^15–18^. We also observed several novel mutational patterns across BCLs that have not been previously reported. In DLBCL, SETD2 mutations were significantly more frequent compared to the rest of the cohort (*P* < 0.001). Mutations in MYC and TBL1XR1 were observed more frequently in BCLs than TCLs, but were not specific to cell size. MEF2C mutations were predominantly found in medium-sized BCLs (*P* < 0.05). TCLs were characterized by frequently mutated SATB1, a transcriptional repressor mainly expressed in thymocytes, exhibiting a mutation rate of 23% (*P* < 0.001) as observed in previous studies^19,20^. The serine and/or threonine protein kinase genes—MTOR and PIK3CD—showed significantly higher mutation rates in CD4+ TCL (*P* < 0.001 and <0.05, respectively).

In our analysis of somatic mutations across canine lymphoma immunophenotypes, several genes emerged as recurrently mutated. Notably, the chromatin remodeling genes KMT2C and KMT2D exhibited the highest mutation rates, detected in 54% and 50% of cases, respectively (Supplementary Fig. 2). These rates align with findings in certain human hematopoietic cancers, i.e., acute myeloid leukemia (AML) and B-cell lymphomas^21^. Other key epigenetic modifiers, namely ARID1A and CREBBP, showed significant mutation rates, 29% and 26%, respectively. SETD2, identified as a putative oncogenic driver in canine cancers, was mutated in 17% of all cases and in 26% of BCL cases, aligning with previous reports^5,22,23^. We also identified several genes with recurrent mutations that, to the best of our knowledge, have not been previously reported in canine cancers. BCL11B, a protein-coding gene implicated as a driver in human T-cell acute lymphoblastic leukemia (T-ALL)^24^, was the third most frequently mutated gene in our analysis, present in 46% of the cohort. The fourth most mutated gene (45%) in our cohort was NOTCH2, which is recognized for its role in human T-cell leukemia^25^.

**Fig. 2:**
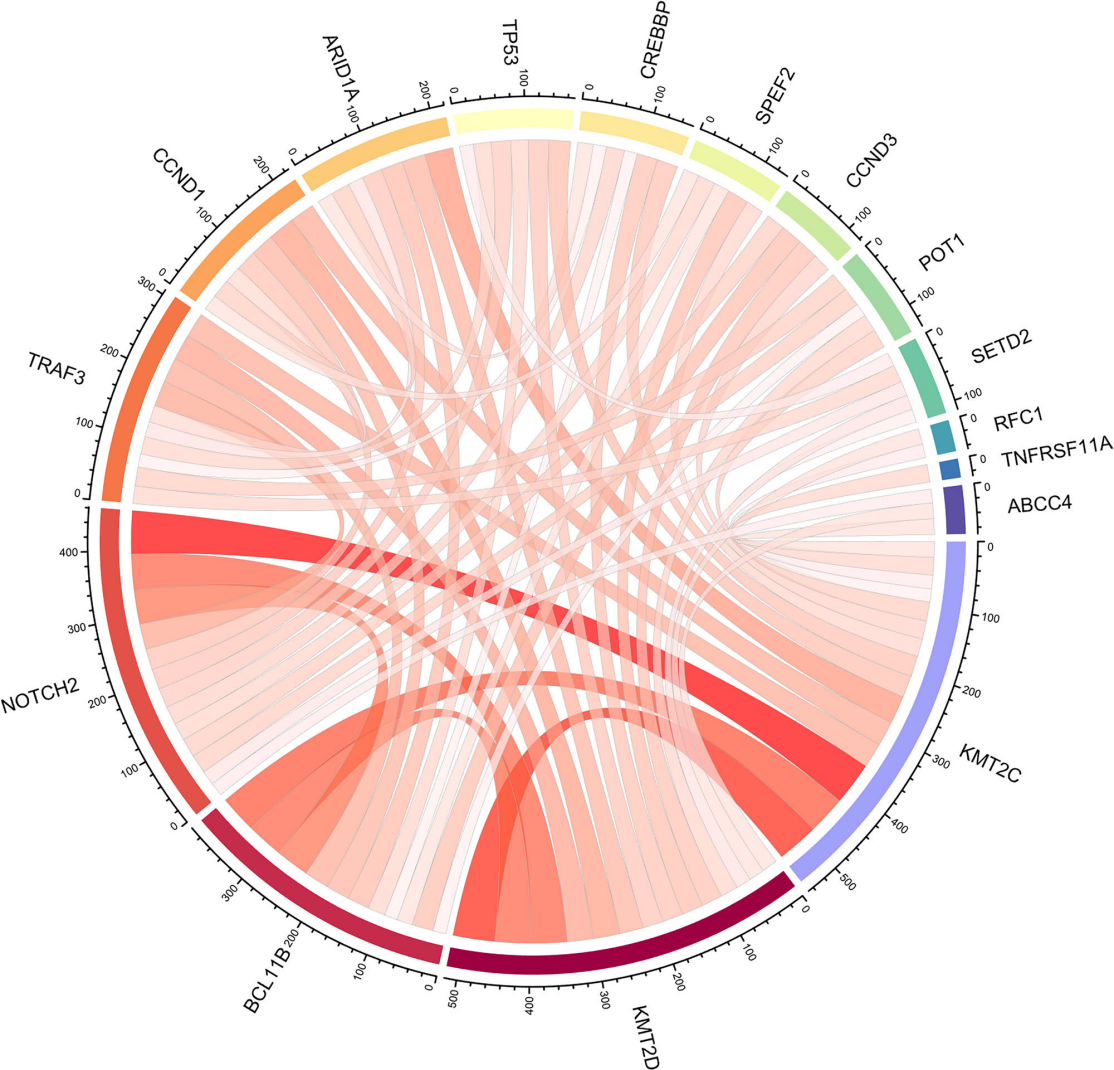
Circos plot depicting co-mutations with greater than 20 co-occurrences among the top somatically mutated genes in our cohort. Outer rings represent the total number of pairwise co-mutations detected. The thickness of the ribbons scales with the total number of co-occurrences observed. Mutual exclusivity and co-occurrence of somatic mutations were assessed by odds ratio.

Missense mutations constituted the majority of the somatic mutations observed across our study. Amongst the top mutated genes, SETD2, TP53, and TRAF3 had the highest frequencies of nonsense mutations: 17%, 13%, and 25%, respectively. Notably, the frequency of nonsense mutations in TP53 closely aligns with the rate reported in a previous study of canine DLBCL^4^. KMT2D and KMT2C exhibited a high incidence of missense mutations, accounting for 64% and 72% of the mutation category in these genes, respectively. This trend was also observed in other highly mutated genes, including TP53, SETD2, FBXW7, and TRAF3. Interestingly, SETD2 and TRAF3 had a higher proportion of frameshift mutations than the other top mutated genes: 17% and 25% respectively (Supplementary Fig. 3). Co-occurrence was the most frequently observed between KMT2C and NOTCH2 (28%), followed by KMT2D and KMT2C (27%), KMT2C and BCL11B (24%), KMT2D and BCL11B (23%), and KMT2D and NOTCH2 (23%) (Fig. 2). In terms of odds ratio (OR), co-occurrence was the most significant between TRAF and SETD2 (*P* < 0.001; OR = 5.7), TRAF3 and POT1 (*P* < 0.01; OR = 4.7), TRAF3 and TBL1XR1 (*P* < 0.05; OR = 6.4), TP53 and SETD2 (*P* < 0.05, OR = 4.2) and POT1 and FBXW7 (*P* < 0.05; OR = 6.6).

### Influence of TRAF3 mutation on clinical outcomes

**Fig. 3:**
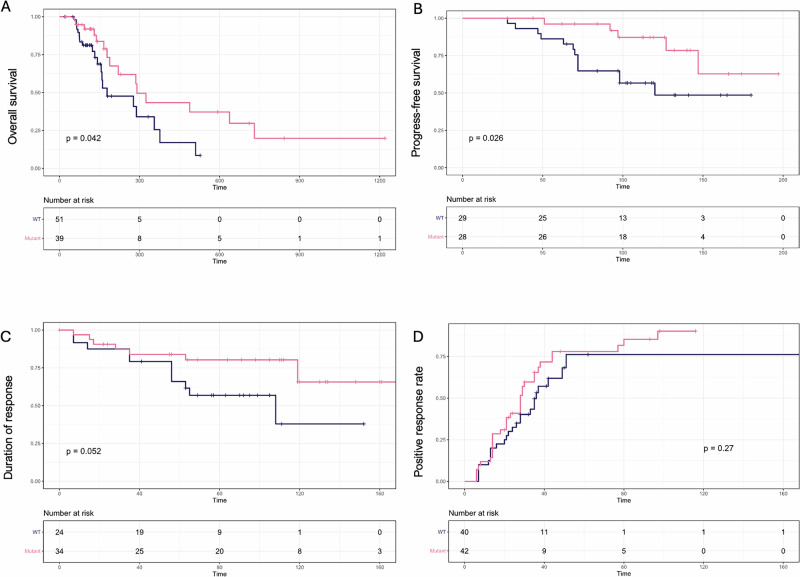
Comparison of clinical outcomes in patients with and without putative somatic, truncating mutations in TRAF3. **A** OS, **B** PFS, **C** DOR, and **D** TTR following the CHOP therapy.

Given the high frequency of somatic mutations in TRAF3 across all immunophenotypes in our study, we assessed the relationship between TRAF3 mutation status and the clinical outcomes of the patients. The distribution of nonsense and frameshift mutations in TRAF3 spanned multiple domains, with the most prevalent nonsense mutation being c.1267C > T, which leads to a truncation of the protein at Arg 423. This mutation, identified in 13 patients, is located at the N-terminal end of the meprin and TRAF homology (MATH) domain, which is crucial for the trimerization of TRAF family proteins^26^. We compared the clinical outcomes of the patients with loss-of-function (LOF) mutations localized to this domain, relative to those patients designated as wild-type (WT) for TRAF3 (Fig. 3). In terms of hazards ratio (HR), Putative LOF mutations in TRAF3 were significantly associated with extended PFS (HR = 0.32, 95% CI of 0.12–0.91) and longer OS (HR = 0.49, 95% CI of 0.24–0.99), and a trend towards a longer duration of response (*P* = 0.052). In contrast, we did not observe a significant association with time to response (TTR). In contrast, neither somatic mutations rated as low, modifier impact or germline truncating variants in TRAF3 were significantly associated with any clinical outcomes (Supplementary Fig. 4).

### Immunophenotype-specific mutations relevant to treatment outcomes

PFS following first-line treatments differed significantly between the two main immunophenotypes, BCLs and TCLs. Moreover, in dogs treated with CHOP, both PFS and OS varied significantly between these two primary immunophenotypes. The median PFS and OS following CHOP treatment were 139 days (95% CI of 112–211) and 290 days (95% CI of 188–488), respectively. BCLs had a superior median PFS of 170 days (95% CI: 127–203) compared to 92 days (95% CI of 84–142) observed for TCLs. These results are consistent with the treatment outcomes previously reported in a retrospective analysis of CHOP chemotherapy^27,28^.

**Fig. 4:**
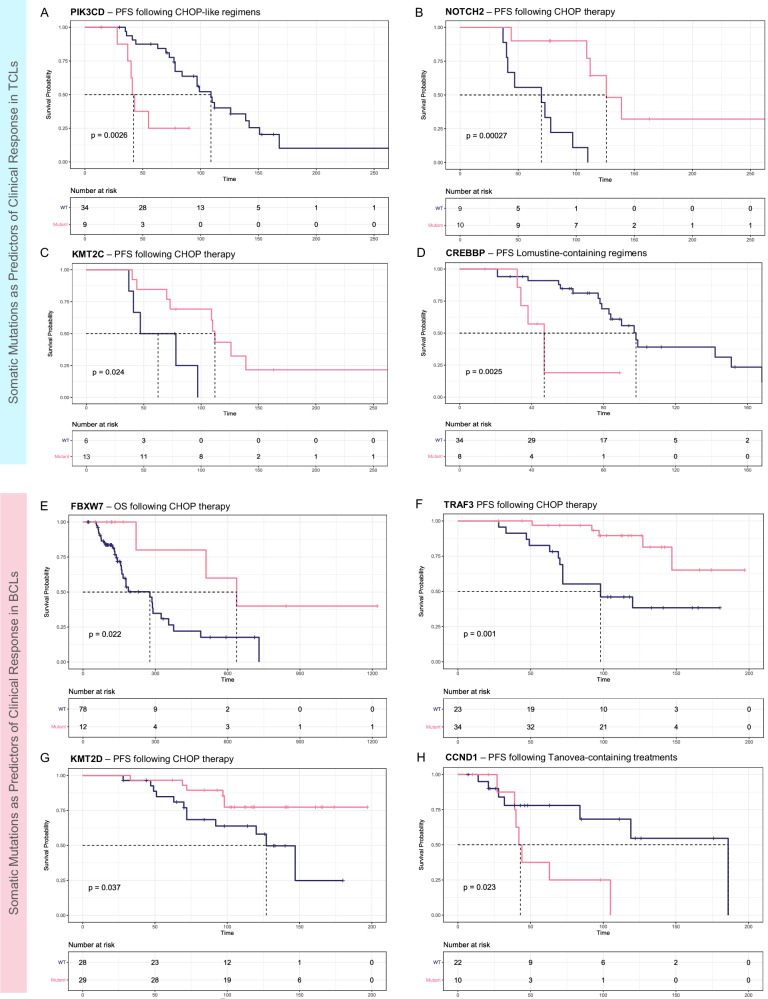
Survival analysis of the BCLs and TCLs with respect to somatic mutation in the influential genes. PFS or OS of the patients following CHOP therapy or treatment via lomustine- or rabacfosadine-containing regimens are compared with respect to mutation in **A** PIK3CD, **B** NOTCH2, **C** KMT2C, and **D** CREBBP for TCLs, and **E** FBXW7, **F** TRAF3, **G** KMT2D, and **H** CCND1 for BCLs.

Given the influence of immunophenotype on survival, we analyzed the influence of the somatic mutations for the BCLs and TCLs separately. Among BCLs, we identified four mutated genes associated significantly with response to CHOP and Tanovea-based regimens (Fig. 4). Specifically, mutated TRAF3 and KMT2D were significantly associated with longer PFS (HR = 0.21, 95% CI of 0.07–0.58; HR = 0.37, 95% CI of 0.14–0.98) while mutated FBXW7 was associated with a significantly extended OS (HR = 0.26, 95% CI of 0.08–0.88) in the patients receiving CHOP therapy. Mutated CCND1 was associated with a notably shorter PFS (HR = 3.6, 95% CI of 1.1–11.4). Among TCLs, we also identified four mutated genes related to response to specific treatment regimens (Fig. 4). Somatically mutated PIK3CD and CREBBP were associated with significantly shorter PFS (HR = 4.2, 95% CI of 1.5–11.7; HR = 4.9, 95% CI of 1.6–14.7) in the patients treated with CHOP or lomustine-containing regimens. Conversely, the patients with mutated KMT2C or NOTCH2 achieved significantly longer PFS following the CHOP treatment protocols (HR = 0.24, 95% CI of 0.06–0.92; HR = 0.09, 95% CI of 0.02–0.43).

The influence of TRAF3, PIK3CD, and CREBBP mutations on survival remained significant during multivariate analysis of each gene, wherein the traditional prognostic factors—immunophenotype, stage, and disease status (naïve vs relapsed)—were included for comparison (Table 2). Immunophenotype was a significant covariate in every per-gene Cox PH model constructed for PFS, except for TRAF3. Treatment status (naïve vs relapsed) was a significant covariate for OS in every model. In addition to the above eight genes, we discovered that the somatic mutation status of TP53 was significantly associated with shorter OS following the first-line therapies.

**Table 2:** Baseline characteristics of the lymphoma cohort

Gene	Covariate	PFS (*N* = 117, events = 41)		OS (*N* = 208, events = 74)	
		HR (95% CI)	*P*-value	HR (95% CI)	*P*-value
PIK3CD	Genotype (MT vs WT)	3.4 (1.4–8.5)	0.0094	0.9 (0.3–2.6)	0.84
	Immunophenotype^a^	2.2 (1.1–4.3)	0.022	1.2 (0.7–2.0)	0.61
	Stage (III–V vs I–II)	0.6 (0.3–1.3)	0.2	1.2 (0.8–2.0)	0.37
	Disease status^b^	1.1 (0.4–3.1)	0.8	2.0 (1.2–3.4)	0.0057
NOTCH2	Genotype (MT vs WT)	0.9 (0.5–1.7)	0.8	0.9 (0.6–1.5)	0.72
	Immunophenotype	2.4 (1.2–4.6)	0.0094	1.1 (0.7–2.0)	0.63
	Stage (III–V vs I–II)	0.64 (0.32–1.3)	0.22	1.3 (0.8–2.1)	0.35
	Disease status	1.05 (0.39–2.83)	0.92	2.0 (1.2–3.4)	0.006
KMT2C	Genotype (MT vs WT)	0.9 (0.5–1.8)	0.84	0.8 (0.5–1.2)	0.23
	Immunophenotype	2.5 (1.3–4.8)	0.0092	1.2 (0.7–2.0)	0.54
	Stage (III–V vs I–II)	0.6 (0.3–1.3)	0.22	1.3 (0.8–2.1)	0.32
	Disease status	1.1 (0.4–3.0)	0.88	2.1 (1.3–3.4)	0.0048
CREBBP	Genotype (MT vs WT)	2.1 (1.1–3.9)	0.026	1.5 (0.9–2.4)	0.15
	Immunophenotype	2.3 (1.2–4.4)	0.015	1.1 (0.7–1.9)	0.65
	Stage (III–V vs I–II)	0.6 (0.3–1.3)	0.19	1.3 (0.8–2.0)	0.34
	Disease status	1.2 (0.4–3.2)	0.75	2.2 (1.3–3.6)	0.0033
FBXW7	Genotype (MT vs WT)	1.0 (0.4–2.5)	0.92	0.5 (0.2–1.2)	0.14
	Immunophenotype	2.4 (1.2–4.6)	0.01	1.1 (0.6–1.8)	0.81
	Stage (III–V vs I–II)	0.6 (0.3–1.3)	0.2	1.3 (0.8–2.0)	0.34
	Disease status	1.1 (0.4–2.8)	0.91	2.1 (1.3–3.5)	0.004
TRAF3	Genotype (MT vs WT)	0.4 (0.1–1.0)	0.049	0.9 (0.5–1.6)	0.7
	Immunophenotype	1.4 (0.7–3.1)	0.39	1.1 (0.6–2.0)	0.79
	Stage (III–V vs I–II)	0.6 (0.3–1.2)	0.17	1.2 (0.8–2.0)	0.38
	Disease status	0.8 (0.3–2.2)	0.62	2.0 (1.2–3.4)	0.0059
KMT2D	Genotype (MT vs WT)	1.0 (0.5–1.8)	0.91	0.8 (0.5–1.2)	0.27
	Immunophenotype	2.4 (1.3–4.6)	0.0086	1.1 (0.7–1.9)	0.67
	Stage (III–V vs I–II)	0.6 (0.3–1.3)	0.2	1.2 (0.7–1.9)	0.53
	Disease status	1.1 (0.4–2.8)	0.91	2.0 (1.2–3.4)	0.0063
CCND1	Genotype (MT vs WT)	1.9 (1.0–3.6)	0.061	1.3 (0.8–2.1)	0.26
	Immunophenotype	2.2 (1.2–4.3)	0.018	1.1 (0.6–1.9)	0.72
	Stage (III–V vs I–II)	0.5 (0.3–1.1)	0.093	1.2 (0.7–1.9)	0.5
	Disease status	0.9 (0.3–2.6)	0.89	2.0 (1.2–3.3)	0.0081
TP53	Genotype (MT vs WT)	1.9 (0.9–4.1)	0.12	2.7 (1.7–4.4)	<0.0001
	Immunophenotype	2.5 (1.3–4.9)	0.0058	1.2 (0.7–2.0)	0.58
	Stage (III–V vs I–II)	0.6 (0.3–1.2)	0.15	1.3 (0.8–2.1)	0.32
	Disease status	1.1 (0.4–2.9)	0.89	1.8 (1.1–3.0)	0.022

^a^Non-T zonal TCLs vs BCLs.

^b^Relapsed vs naïve.

## Discussion

Our study represents one of the largest tNGS-based studies of canine lymphoma to date, involving an analysis of the clinical, genetic, and phenotypic data of 238 dogs with spontaneously occurring lymphoma for 308 genes. We discovered several significant associations between immunophenotypic subtypes, treatment outcomes, and specific gene alterations, with some showing promise as potential therapeutic targets. We identified eight genes—TRAF3, FBXW7, KMT2D, CCND1, PIK3CD, NOTCH2, KMT2C, and CREBBP—in which somatic mutations may serve as potential novel biomarkers of clinical response. By focusing our univariate survival analysis specifically on somatic mutations within distinct immunophenotypes, we sought to identify driver mutations rather than incidental passenger mutations. This methodological choice also allowed for a more controlled analysis, accounting for the variability in survival outcomes attributable to differences in immunophenotypes.

TRAF3, which emerged as one of the top somatically mutated genes in BCLs in our study, has been recognized as a potential driver of B-cell lymphomagenesis in both canine and human studies^5,7^. As a known negative regulator of the non-canonical NF-κB pathway, TRAF3 is a putative tumor suppressor in the realm of human and translational research^29,30^. We also observed that somatic LOF mutations in TRAF3 are almost exclusively seen in BCLs and positively associated with longer OS and PFS. These findings suggest that TRAF3-mutated BCLs have an increased sensitivity to conventional chemotherapeutics. In contrast, the function of NF-kB signaling affects only the late-stage differentiation of CD4+ T-cells^31^. Interestingly, in human DLBCL models, deletion of the TRAF3 gene induces reliance of cancer cells on non-canonical NF-κB signaling for survival^32^. This pathway dependency may render cells more vulnerable to disruption by DNA alkylators such as doxorubicin and cyclophosphamide, both components of CHOP therapy. If this mechanism is conserved in canine lymphoma, it may also highlight components of the non-canonical NF-κB pathway as potential therapeutic targets.

PIK3CD and CREBBP emerged as potential biomarkers for inferior outcomes in TCLs, specifically associated with shorter PFS in CHOP-like treatments and CCNU-containing treatments, respectively. PIK3CD encodes an isoform of the phosphoinositide 3-kinase (PI3K) enzyme, an integral component of the PI3K/AKT/mTOR pathway. Notably, novel therapeutics are under development to target components of this pathway in human oncology^33,34^. PIK3CD promotes the activation of c-MYC and p-AKT, both of which drive cell proliferation and survival^35^. Mutations in PIK3CD may therefore lead to constitutive survival signaling, which in turn may reduce the efficacy of DNA-damaging agents such as doxorubicin and cyclophosphamide—both key components of CHOP—by promoting cell survival and impairing apoptosis in response to chemotherapy. PIK3CA has been previously linked to shorter PFS in a canine pan-cancer study^36^. PIK3CA mutations are also associated with inferior prognosis in triple-negative breast cancer^37^, and PIK3CD mutations have been implicated as potential indicators of poor prognosis in breast carcinoma. On the other hand, CREBBP encodes a histone acetyltransferase that functions as a transcriptional co-activator and chromatin modifier. LOF mutations in CREBBP can impair histone acetylation, leading to a more condensed chromatin state and transcriptional repression of tumor suppressor genes, contributing to lymphomagenesis^38,39^. The efficacy of alkylating chemotherapeutics such as lomustine can be diminished as tumors with CREBBP loss may have enhanced capacity to maintain chromatin compaction or evade apoptosis despite DNA damage. Histone deacetylase inhibitors (HDACi) have been shown to sensitize tumor cells to DNA-damaging agents by disrupting chromatin structure and impairing access to DNA repair machinery^40^. In this regard, CREBBP-deficient TCLs may be less responsive to lomustine-based therapies unless paired with an epigenetic sensitizer.

Other putative prognostic biomarkers in TCLs identified in our study include NOTCH2 and KMT2C. NOTCH2 is implicated in T-cell lineage commitment, while KMT2C plays a role in epigenetic regulation. However, the impact of these genes on cancer prognosis remains less understood and is a subject for further investigation. Interestingly, somatic mutations in both KMT2D and KMT2C were associated with longer PFS in BCLs and TCLs, respectively. KMT2 family genes have been cited as some of the most highly mutated genes across several large-scale sequencing studies in human hematopoietic cancers^41^. While the roles of these epigenetic regulators in oncogenesis are well established, the association of their mutational status with prognosis remains inconsistent. LOF mutations in KMT2C and KMT2D have been associated with improved outcomes in pancreatic ductal adenocarcinoma, whereas in mantle cell lymphoma and non-small cell lung cancer, KMT2D mutations have been linked to poorer survival^42,43^. These discrepancies may reflect differences in tissue-specific enhancer programs or the differentiation state of the tumor, factors that influence whether KMT2D loss disrupts oncogenic or tumor-suppressive transcriptional programs^34^. Further research is needed to understand the role of these genes in canine lymphoma, although our findings point to a shared potential of KMT2 genes as predictors of positive outcome.

Other epigenetic regulators found to have significant associations in our cohort included SETD2, more frequently mutated in DLBCLs, and SATB1, frequently mutated in TCLs. Somatic SETD2 mutations have been recurrently reported in canine DLBCLs and, in some studies, associated with shorter PFS^20^. In humans, SETD2 encodes a histone methyltransferase responsible for H3K36 trimethylation, a mechanism critical for recruiting double-stranded DNA break repair machinery. Loss of function can lead to increased mutational burden and genomic instability, and is theorized to contribute to tumorigenesis, particularly in B-cell lymphocytic leukemias^44^. However, the prognostic relevance of SETD2 mutations remains inconsistent across studies in canine lymphoma, and in our cohort, they were not significantly associated with PFS^45^. Further research is needed to clarify whether SETD2 functions similarly in canine lymphoma. SATB1, a known chromatin organizer, plays a key role in T-cell differentiation and has been implicated in the pathogenesis of human cutaneous T-cell lymphoma. Its frequent mutation in our TCL cohort may reflect dysregulation of these pathways^46^.

Our analysis of 238 canine samples was broadly representative of the immunophenotypic distribution observed in clinical practice, with 64% of dogs diagnosed with BCL and 31% with non-TZL TCL, compared to 71% and 20%, respectively, in a U.S. study of canine nodal lymphomas^47^. Among our TCL cases, 60 of 83 were classified as CD4+, consistent with the 72% CD4+ prevalence reported for non-TZL TCLs in the same study. As a result, the prognostic utility of the genotypes identified here is largely reflective of this subtype, which is associated with significantly longer OS than its CD8+ or double-negative counterparts. While these findings are valuable, they also highlight the limitations imposed by the phenotypic heterogeneity of TCLs. The significance of the biomarkers reported here may not be generalizable to less common T-cell subtypes. There is a significant need for research to evaluate genetic biomarkers within a broader spectrum of TCL subtypes in dogs. A future study in an expanded cohort of TCLs, including CD8+, double-positive, double-negative, and T-zone lymphoma, could provide further insight into the mutational landscape and prognostic value of genetic biomarkers found within these subtypes. In addition, breed-specific predispositions to lymphoma subtypes have been reported, but our univariate analyses were not stratified by breed to maintain sufficient case numbers per treatment group. Validation in a breed-controlled cohort would help assess the generalizability of our findings.

Our study focused on gene-level associations with immunophenotype and clinical outcome to offer greater statistical power and interpretability of the results. A follow-up study in a larger cohort would enable variant-level association testing and improve the ability to detect and validate statistically significant hotspot mutations. This, in turn, could guide the design of functional studies to better understand the mechanisms underlying both sensitivity and resistance to chemotherapy in canine lymphoma. Functional validation of the putative loss-of-function mutations identified in this study, particularly in TRAF3, would help confirm causality and clarify their therapeutic relevance. This could include in vitro gene knockout experiments to assess sensitivity to CHOP agents, or ex vivo drug sensitivity testing using patient-derived cells with and without the reported TRAF3 variants.

Given that driver mutations are typically somatic, matched germline samples were included for variant calling. However, the use of whole blood as normal tissue in a systemic malignancy such as multicentric lymphoma presents limitations. To reduce the likelihood of false-positive somatic calls, we applied a conservative allele frequency threshold of >0.05 in the matched blood samples. Thus, it is possible that true somatic variants present at low frequencies in blood due to the presence of circulating tumor cells may have been excluded. Incorporating a panel of normal samples from healthy dogs and application of a lower allele frequency threshold would improve the robustness of somatic variant detection. Our analysis did not include an assessment of copy number alterations (CNAs), which are known to contribute to the pathogenesis of several canine cancers. Future studies validating our initial findings in whole-exome or whole-genome sequencing data would also be better suited to assess CNAs in canine lymphoma.

In conclusion, our findings provide significant insights into the mutational landscape of canine lymphoma. This research, encompassing 238 dogs, reveals critical correlations between genetic mutations and clinical outcomes in various chemotherapeutic regimens. The identification of recurrent mutations in genes such as KMT2C, KMT2D, NOTCH2, TRAF3, CCND1, ARID1A, CREBBP, and TP53, with their varied implications in both BCLs and TCLs, highlights the potential of genomic profiling to inform future treatment strategies. While additional validation is needed to support clinical implementation, these findings represent an important step toward realizing precision medicine in veterinary oncology.

## Methods

### Patient selection and immunophenotyping

The patient selection criteria were defined as follows. Dogs diagnosed with lymphoma by cytology, confirmed by a board-certified veterinary pathologist, and treated at veterinary oncology clinics between 2017 and 2023 were included in the study (*N* = 4256). From this population, a subgroup of 248 dogs met the inclusion criteria for the study: (1) Availability of matching tumor and normal specimens; (2) both immunophenotyping and ex vivo drug sensitivity data generated; (3) documented clinical response to at least one well-established chemotherapy regimen within a month following biopsy sampling. Ten dogs were excluded from analysis due to poor sequencing quality, leaving a cohort of 238 dogs for analysis. All specimens were collected under informed consent, and the experiments were approved by the internal review board (IMVLSA1223.18), as well as the ethics committee of the participating veterinary hospitals.

To group the cohort into clinically relevant immunophenotype subtypes, immunophenotyping was conducted using a combination of flow cytometry (FC) and PCR for antigen receptor rearrangement (PARR) as previously described^48^. Briefly, BCLs were categorized by the expression of the mature B-cell marker CD21, while TCLs were identified by the expression of mature T-cell markers CD3, CD4, CD5, and CD8. Lineage confirmation of the samples was achieved through PARR. FC analysis further divided the cohort into nine subtypes. BCLs were classified by relative cell size—small, medium, or large (DLBCL). TCLs were further subtyped based on surface antigen expression: helper-like CD4+ T-cells (CD3+/CD4+/CD8-), CD8+ cytotoxic-like T-cells (CD3+/CD4-/CD8+), CD45- T-zone lymphoma (TZL), and cases with atypical patterns of antigen expression. These atypical presentations included uncommon phenotypes such as double-negative (CD3+/CD4-/CD8-), double-positive (CD3+/CD4+/CD8+), and aberrant phenotypes lacking CD3 expression (CD3-/CD4+/CD8- and CD3-/CD4-/CD8-/CD5+). Due to the indolent disease progression of TZL in canine lymphoma, this subtype was separated from the TCLs during survival analysis.

### Gene selection and tNGS library preparation

Fine needle aspirates (FNAs) of affected lymph nodes were processed by ImpriMed, Inc. (Mountain View, CA, USA) as previously described^43^. Matching whole blood samples, representing normal patient cells, were also provided with the tumor biopsy. Genomic DNA was extracted from FNA and whole blood cells, sheared to a median insert size of 300 bp and prepared using the Enzymatic Fragmentation v2.0 NGS library preparation kit (Twist Bioscience, CA, USA), and barcoded using the Unique Dual Index plates (A-D). The 308-gene panel used in this study was curated based on relevance to lymphoma biology, drug response, and mutational recurrence in both canine and human cancers. Gene inclusion was guided by prior reports in canine lymphoma, entries from the COSMIC database, literature linking genes to chemoresistance or disease progression, and membership in known oncogenic pathways^10^. For genes identified in human studies, known canine orthologs were included when available, using annotations from COSMIC and Ensembl. The capture and enrichment of targeted genes was achieved using the Fast Hybridization kit and a custom probe panel of 308 genes spanning 0.98 Mbp, spread over 4667 regions. Whole genome and post-capture libraries were quantified by the Qubit Flex Fluorometer (Life Technologies, CA, USA). Quality was assessed using the Tapestation 6800 (Agilent, CA, USA). Target-enriched libraries were sequenced on either the Illumina HiSeq 4000 or Illumina NovaSeq 6000 system with 2 × 150 bp paired-end reads.

### Gene alignment and quality control

All targeted sequencing reads were passed through FastQC (v0.11.9) before and after adapter trimming to obtain baseline sequencing quality metrics, including quality scores, GC content, and adapter content. Adapter sequences and low-quality bases were trimmed from the reads using BBDuk (v38.95). Trimmed reads were aligned to the CanFam 3.1 reference genome, sourced from the Ensembl database with BWA-MEM (v0.7.17). Aligned reads were preprocessed for variant calling by harmonization between matched tumor and normal samples using Samtools (v1.9) and deduplication using Picard MarkDuplicates (v2.22.3). Qualimap (v2.2.1) was used to conduct quality control analysis on the preprocessed bam files.

### Variant calling and annotation

Both somatic and germline variants were called with VarDict (v4.3), using the pre-built CanFam3.1.86 database with default parameters as previously described^49^. Briefly, variants with lower than 5× read coverage, mean base quality <22.5, and a variant allele frequency <0.05 were excluded. False positives were excluded by filtering out variants with mean mismatches in reads ≥5.25. To account for the potential presence of a neoplastic cell fraction in peripheral blood, somatic calls were excluded if its harmonized sequence in the normal sample had an allele frequency >0.05. Called mutations included SNVs and small INDELs. Functional variant annotation was performed using a combination of SnpEff and SnpSift (v4.3)^50,51^. SnpEff was used for primary annotation of a variant’s IMPACT ratings, protein amino acid level alterations, and associated transcript Ensembl IDs and Uniprot accession numbers. SnpSift was used to obtain Sorting Intolerant from Tolerant scores for missense variants. Annotated Variant Call Format (VCF) files were used for all downstream analysis. RStudio (v4.3.0) was used to extract mutational features from VCF files for survival analysis and subtype correlation, using the VariantAnnotation, GenomicRanges, and Rsamtools packages^52–54^. Deleterious mutations were filtered based on their IMPACT rating as defined by the Ensembl Variant Effect Predictor. Mutations with moderate and high impact, as determined by sequence ontology, were included in the survival analysis and for comparing mutation frequencies between groups defined by baseline clinical and molecular characteristics. For univariate survival analysis focusing on putative LOF mutations, frameshift variants, stop lost, start lost, stop gained, and missense variants with a SIFT score <0.05 were included.

### Clinical data collection and curation

Longitudinal clinical data were generated based on the medical charts provided by the participating veterinarians. A contracted medical professional, well-versed in clinical annotation, was responsible for the collation of these records. Date and treatment response were documented for every clinical visit before and after the biopsy sampling date. Treatment responses were categorized into progressive disease, stable disease, partial response (PR), or complete response. Additionally, dates of euthanasia or cessation of treatment were recorded. Time to initiation of and clinical response to treatment was noted relative to the sampling date. Using the collated data, we computed PFS and OS according to VCOG v1.0 guidelines, as well as duration of response (DOR) and TTR for each patient across the treatment regimens they received^55,56^. PFS was defined as the time interval from the initiation of treatment to the first observation of disease progression. OS was determined as the time from the start of treatment to the date of death or last follow-up. DOR was defined as the time interval from the first instance of a positive response (PR or better) to the first incidence of disease progression, while TTR was defined as the time from the start of treatment to the first instance of positive response.

Chemotherapy regimens were categorized into CHOP, MOPP, LOPP, other combinations, and single or double agents by a previously described methodology^57^. CHOP-based treatments were defined as at least one single cycle of cyclophosphamide, vinca alkaloids, antitumor antibiotics (doxorubicin or mitoxantrone), and corticosteroids, with or without L-asparaginase induction. COP protocols excluded antitumor antibiotics and were included in the CHOP treatment category unless otherwise specified. MOPP treatments were defined as one cycle of mechlorethamine, vinca alkaloids, procarbazine, and corticosteroids, while LOPP treatments included CCNU in lieu of mechlorethamine with or without L-asparaginase induction. Single and double-agent therapies used one or two drugs, respectively, with or without corticosteroids. Protocols not fitting these classifications were grouped as other combinations.

### Statistical and survival analysis

Baseline clinical and molecular characteristics were compared using the chi-square test or Fisher’s exact test for categorical variables, and a two-sample *t*-test or Mann–Whitney U test for continuous variables. Co-occurrence and mutual exclusivity of variants were analyzed using odds ratios, with the significance of the relationships evaluated by Fisher’s exact test or the chi-square test. *P*-values were corrected using Bonferroni method when multiple tests were indicated. OS and PFS were estimated using the Kaplan–Meier method. Hazard ratios and their 95% CIs were estimated using Cox proportional hazards models. The impact of somatic mutations on PFS, OS, DOR, and TTR was analyzed using both multivariate and univariate models. Non-parametric log-rank tests were applied to compare survival outcomes.

## Supplementary information

20250418_Supporting Information genotyping revision

## Data Availability

The datasets generated and/or analyzed during the current study are not publicly available due to privacy and security concerns, but are available from the corresponding author upon reasonable request.
