# Supplementary material for: Identification of novel genetic mutations for the treatment prognostication of canine lymphoma
Source: NPJ Precis Oncol. 2025 Jun 12;9:174. doi: 10.1038/s41698-025-00988-5 (PMC12162864; doi:10.1038/s41698-025-00988-5)
Supplement: Supplementary file 1 — 20250418_Supporting Information genotyping revision [file 41698_2025_988_MOESM1_ESM.docx]

**Supporting Information for**

Identification of novel genetic mutations for the treatment prognostication of canine lymphoma

Josephine Tsang1, Qi Jing Yap1, Sheena Kapoor1, Jerry Cromarty1, Sushmita Sen1, Minji Kim2,+, George Courcoubetis1,+, Suhyeon Cho1, Deanna Swartzfager1, Stanley Park1, Sungwon Lim1,2, Ilona Holcomb1,*, Jamin Koo1,2,3,*

1ImpriMed, Inc., 1130 Independence Ave, Mountain View, CA 94043, United States

2ImpriMedKorea, Inc., Seoul 03920, Republic of Korea

3Department of Chemical Engineering, Hongik University, Seoul 04066, Republic of Korea

+These authors contributed equally to this work.

Co-correspondence:

Ilona Holcomb (iholcomb@imprimedicine.com) and Jamin Koo (jaminkoo@alumni.stanford.edu)


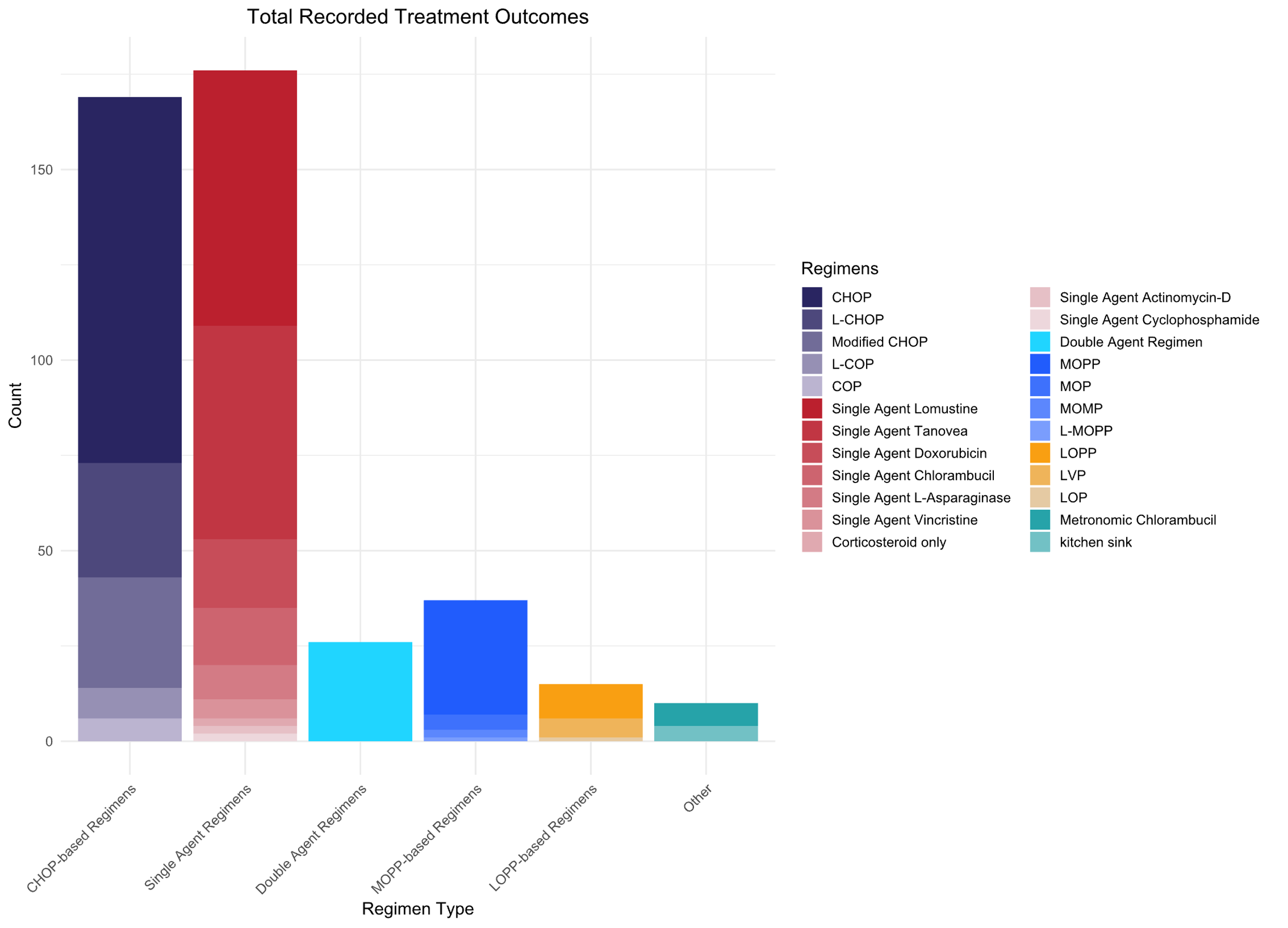

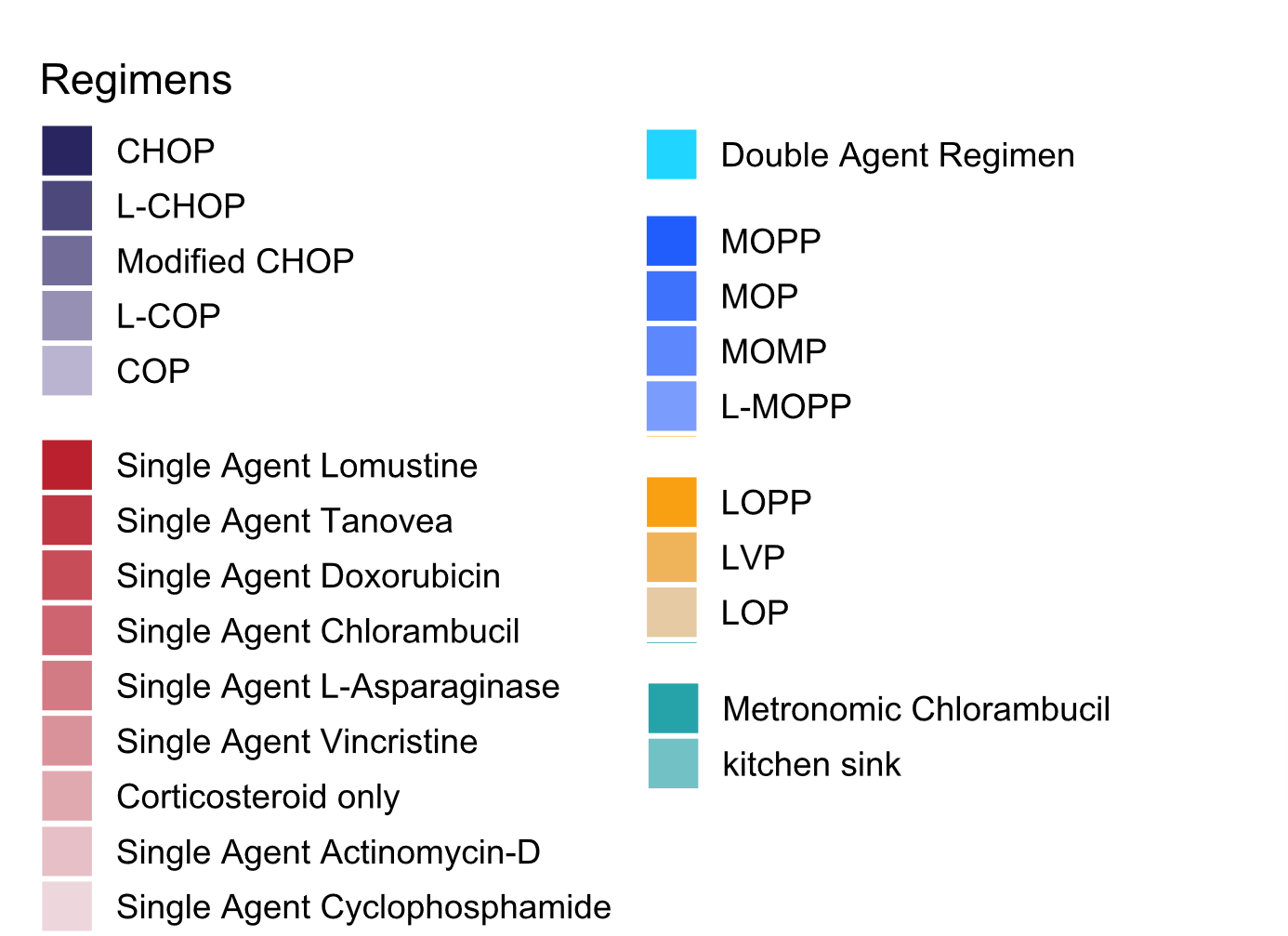


Regimen Type

n=169

n=176

n=26

n=37

n=15

n=10

**Supplementary Fig. 1.** Distribution of recorded treatment regimens and corresponding outcomes among the entire cohort (N=238). For some patients, there were multiple treatment outcomes available across different time points, i.e., t = 4 weeks or 8 weeks.


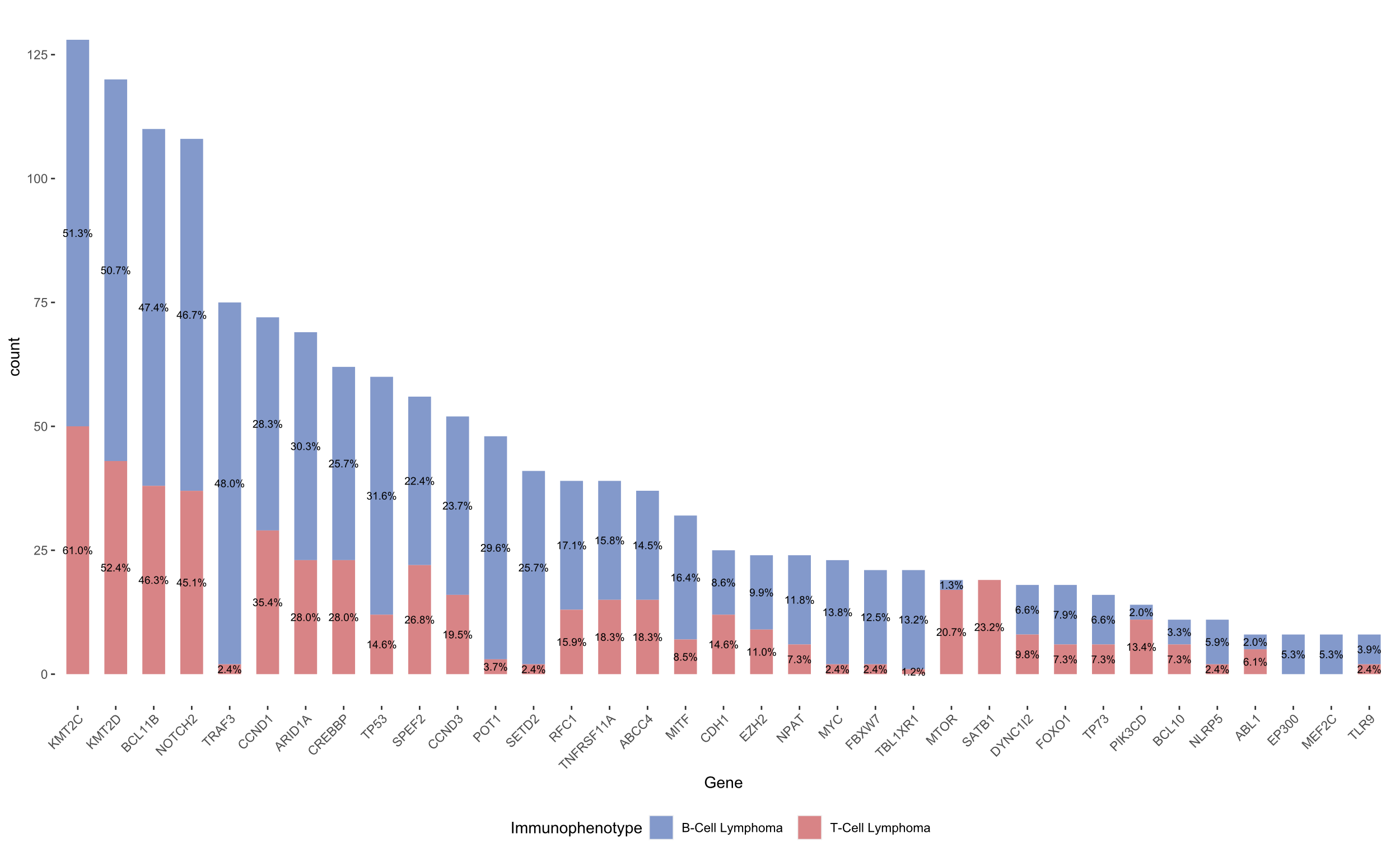


**Supplementary Fig. 2.** Number of patients within the cohort with somatic mutations detected in the top 35 mutated genes. Percentages represent proportion of patients in each immunophenotype group with the gene somatically mutated.

**Supplementary Fig. 3.** Lollipop plot representation of mutational distributions of top mutated genes in our study—(A) KMT2D, (B) BCL11B, (C) SETD2, (D) FBXW7, (E) TP53, and (F) TRAF3. Each colored node represents a variant detected within the gene, with the x-axis showing the corresponding amino acid position, and the y-axis indicating the number of patients harboring that specific variant in our cohort.

**
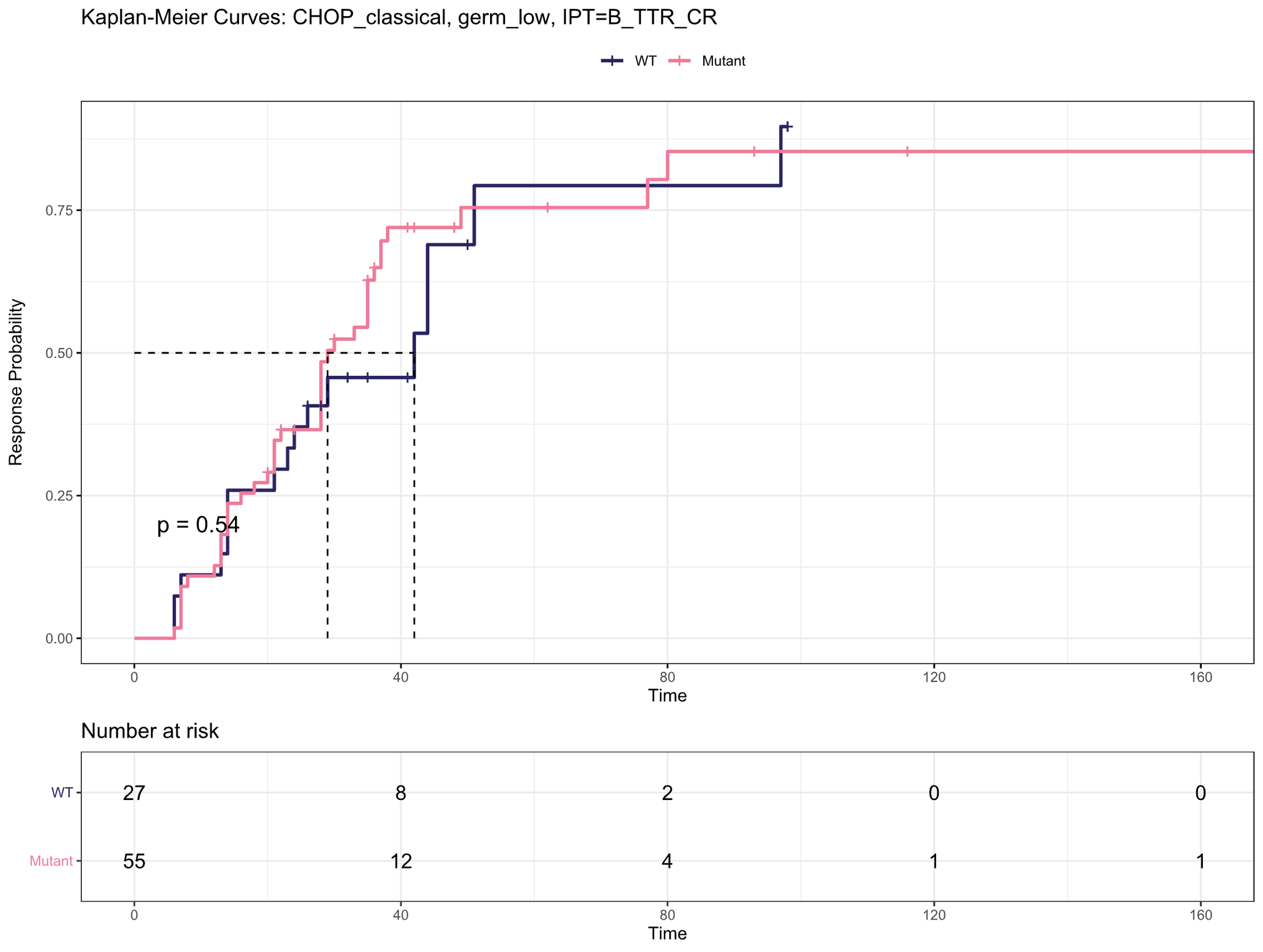

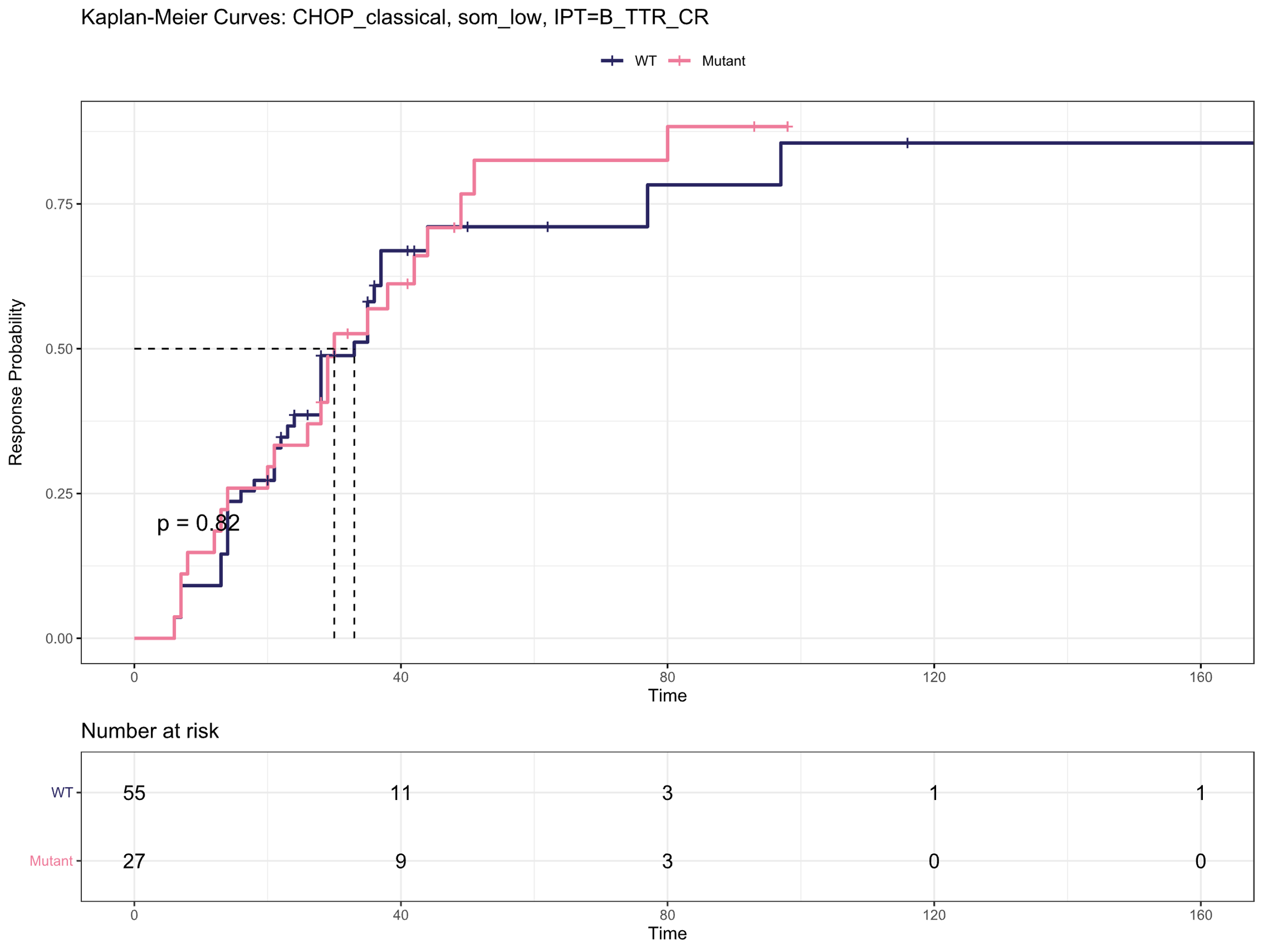

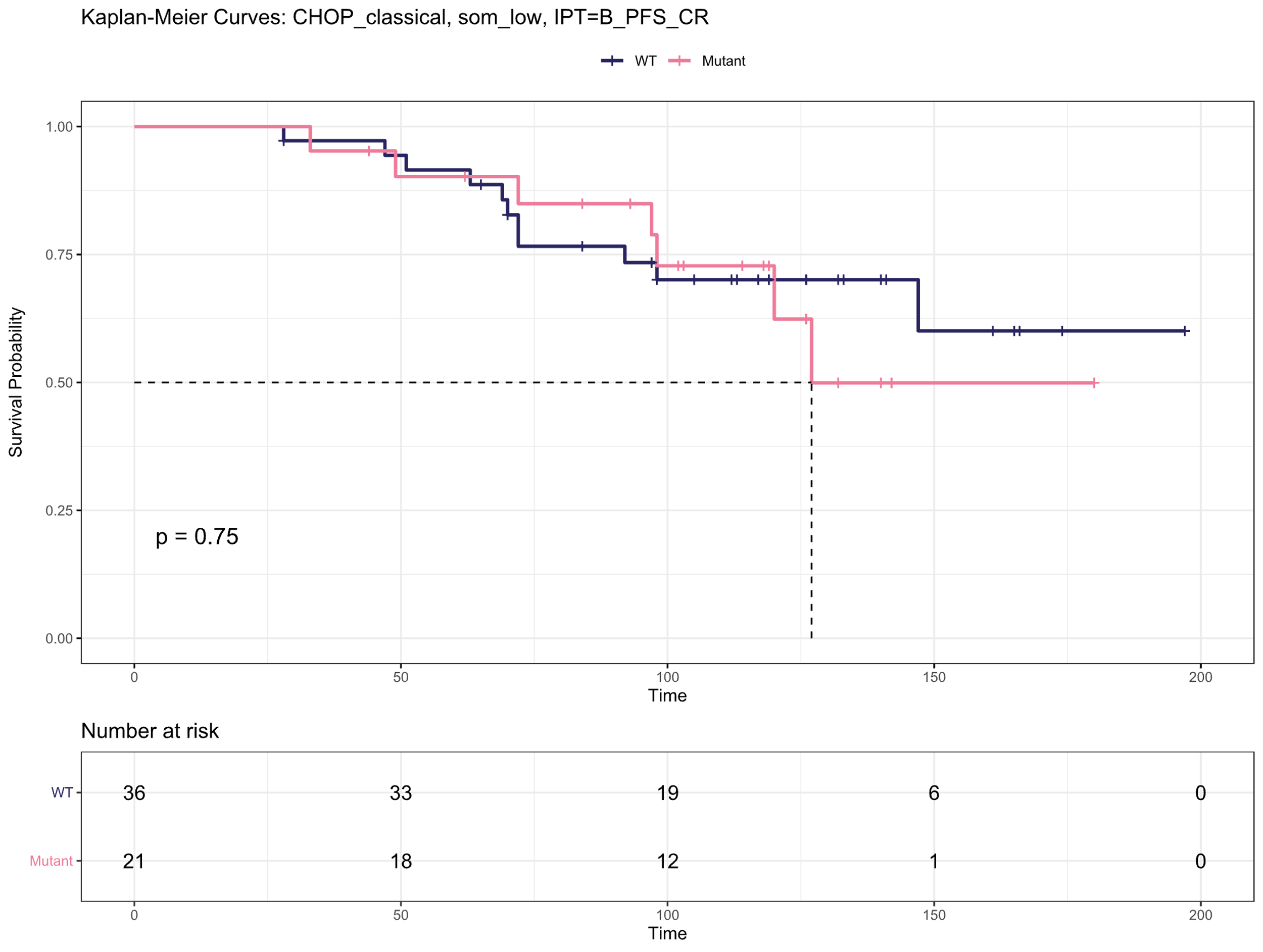

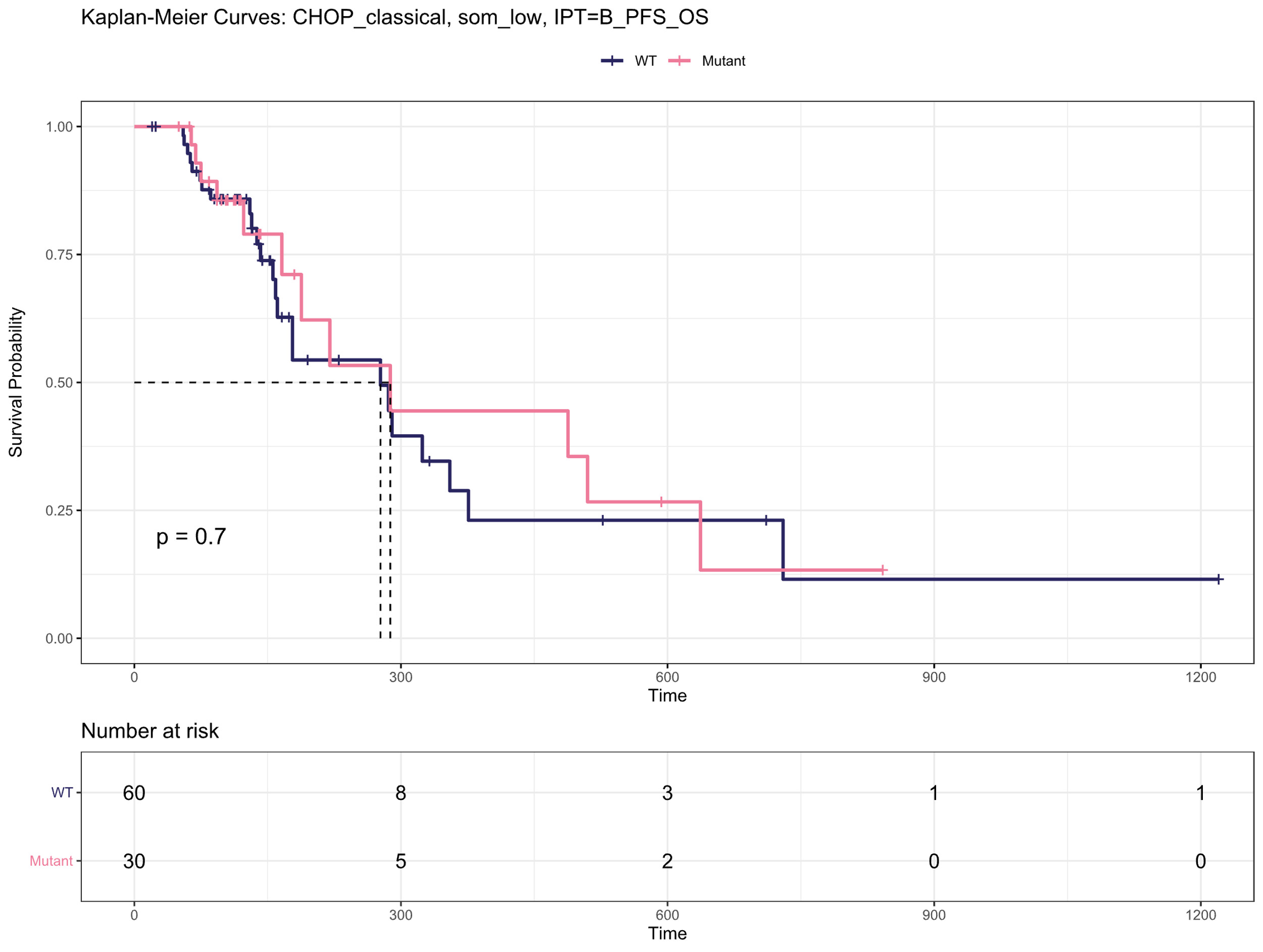

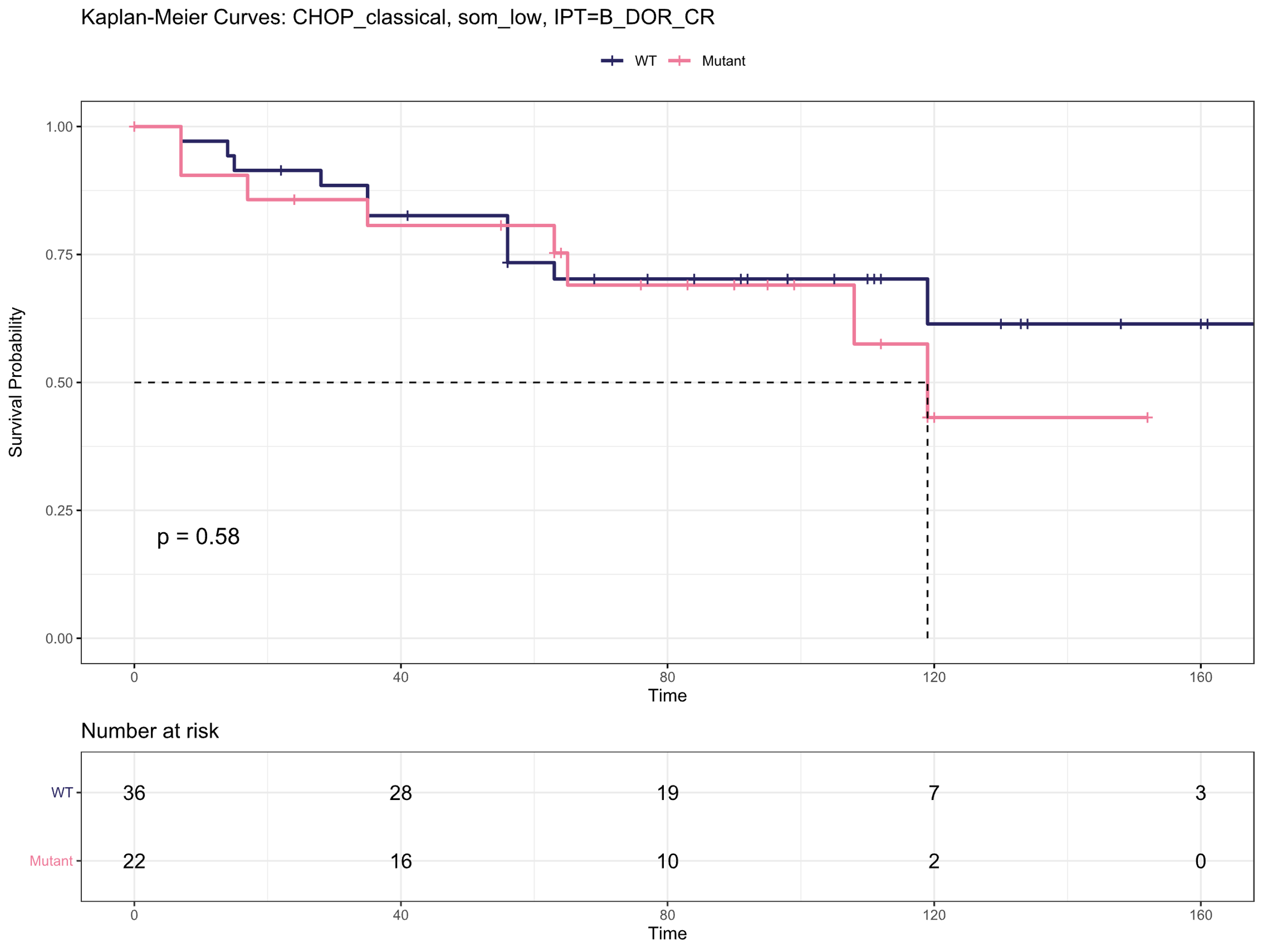

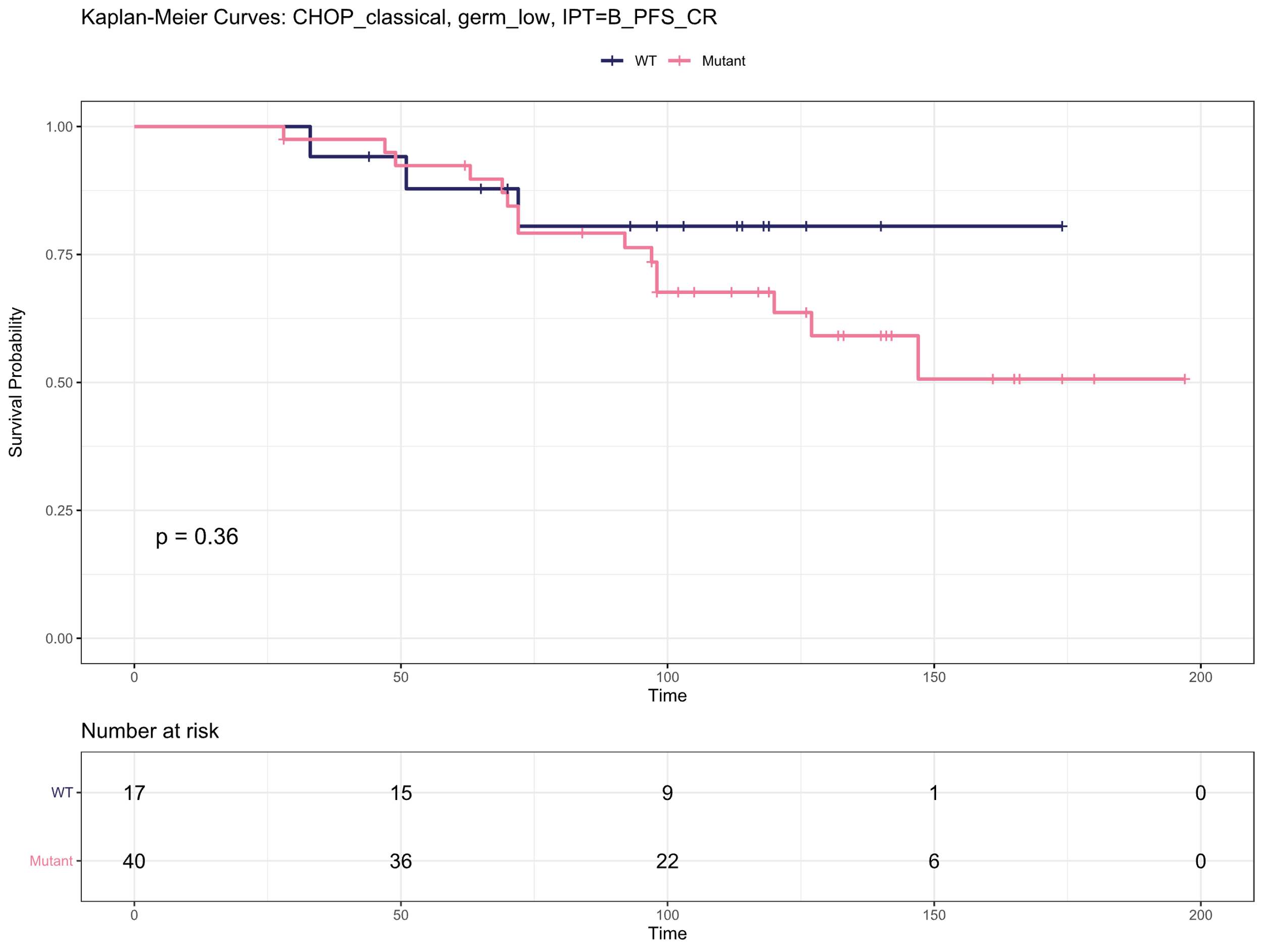

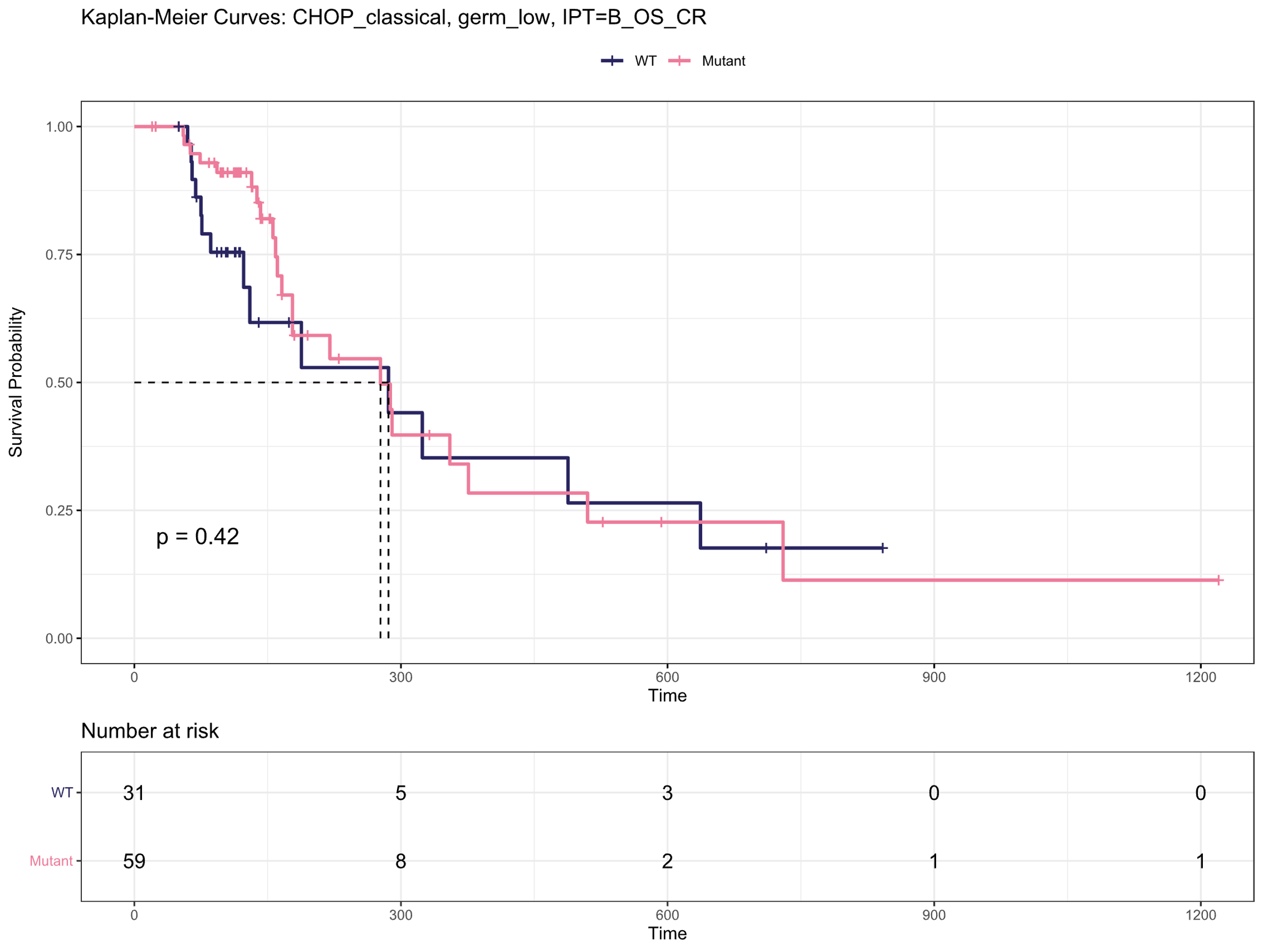

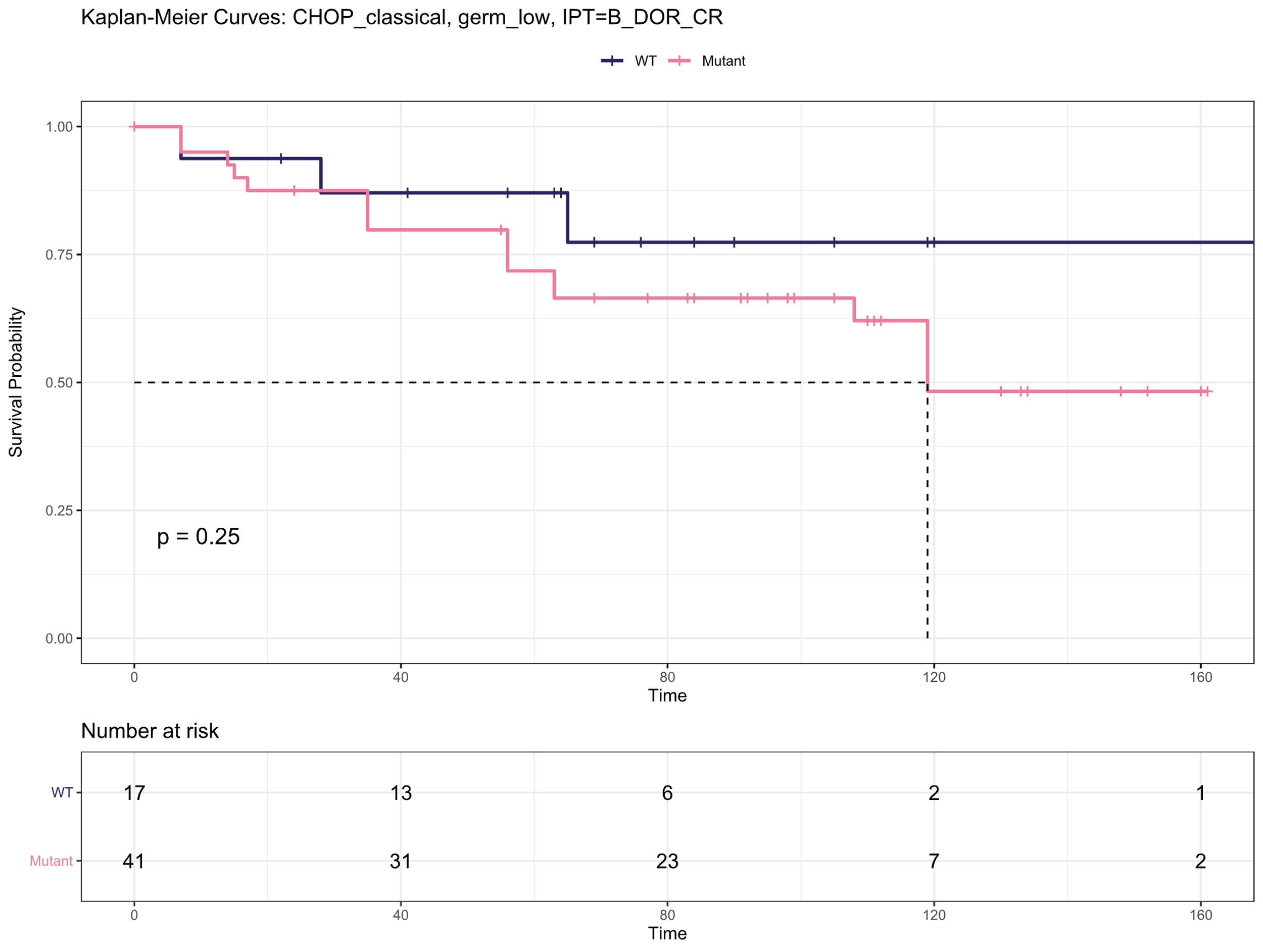
**

**Overall Survival**

**Progression Free Survival**

**Duration of Response**

**Time to Response**

**Overall Survival**

**Progression Free Survival**

**Duration of Response**

**Time to Response**

**Somatic**

**Germline**

A

B

C

D

E

F

G

H

**Supplementary Fig. 4.** Kaplan Meier curves of PFS, DOR, TTR, and OS for CHOP/L-CHOP therapy in TRAF3 for somatic and germline low impact and modifier class mutations.


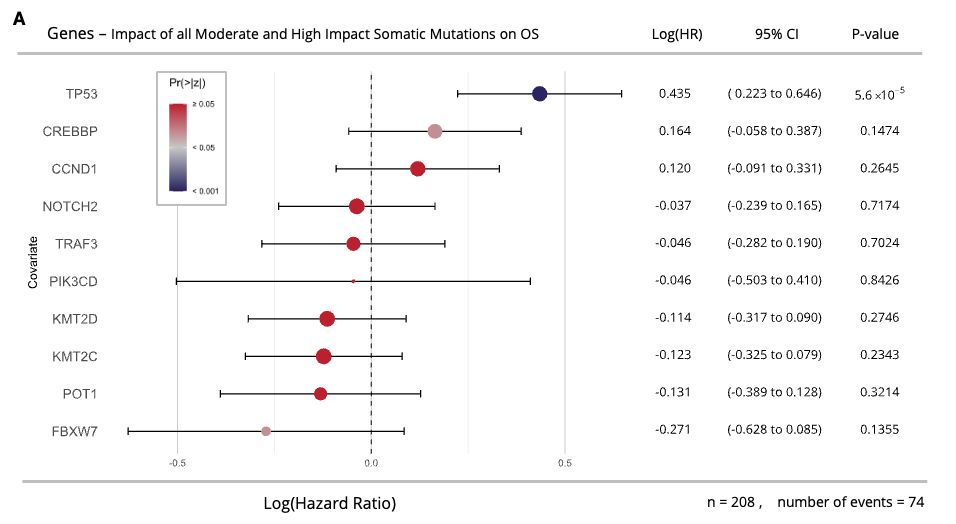


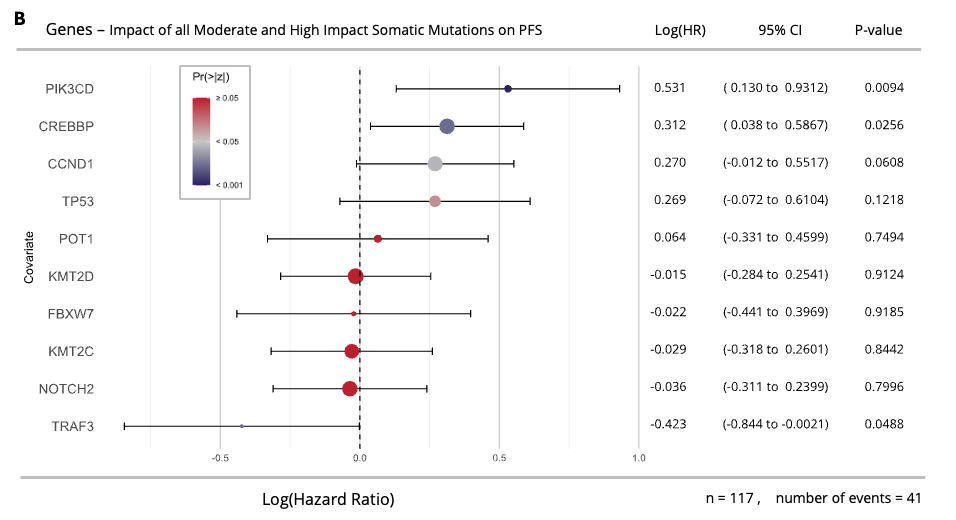


**Supplementary Fig. 5**. Cox proportional hazards forest plot for (A) OS and (B) PFS following first line treatment in 208 patients, with respect to somatic mutational status of each gene.

| Breed | Count | Percentage (N=238) |
| --- | --- | --- |
| Labrador Retriever | 30 | 12.6% |
| Boxer | 26 | 10.9% |
| Golden Retriever | 25 | 10.5% |
| Pit Bull Terrier | 23 | 9.7% |
| German Shepherd Dog | 9 | 3.8% |
| Beagle | 6 | 2.5% |
| American Staffordshire Terrier | 4 | 1.7% |
| Bichon Frise | 3 | 1.3% |
| Bulldog | 3 | 1.3% |
| Collie | 3 | 1.3% |
| Doberman Pinscher | 3 | 1.3% |
| Rhodesian Ridgeback | 3 | 1.3% |
| Shih Tzu | 3 | 1.3% |
| Vizsla | 3 | 1.3% |
| American Bulldog | 2 | 0.8% |
| Australian Cattle Dog | 2 | 0.8% |
| Border Collie | 2 | 0.8% |
| Bullmastiff | 2 | 0.8% |
| Cavalier King Charles Spaniel | 2 | 0.8% |
| Chow Chow | 2 | 0.8% |
| Cocker Spaniel | 2 | 0.8% |
| Corgi | 2 | 0.8% |
| English Springer Spaniel | 2 | 0.8% |
| Greyhound | 2 | 0.8% |
| Havanese | 2 | 0.8% |
| Pomeranian | 2 | 0.8% |
| Portuguese Water Dog | 2 | 0.8% |
| Yorkshire Terrier | 2 | 0.8% |
| Unknown | 23 | 9.7% |
| Other | 38 | 16.0% |
| Mixed Breed | 5 | 2.1% |

**Supplementary Table 1.** Distribution of the top 30 represented breeds in the study cohort. Breed frequencies are shown for the 238 dogs included in the study. The Labrador Retriever, Boxer, and Golden Retriever were the most represented purebred dogs. “Other” includes 38 pure breeds each represented by a single individual. “Unknown” indicates cases with no recorded breed, and “Mixed Breed” includes dogs reported as a mix of two or more breeds.
